# Temperature-Dependent Development of the Two-Spotted Ladybeetle, *Adalia bipunctata*, on the Green Peach Aphid, *Myzus persicae*, and a Factitious Food Under Constant Temperatures

**DOI:** 10.1673/031.010.12401

**Published:** 2010-08-03

**Authors:** Mohammad. Amin. Jalali, Luc Tirry, Abbas Arbab, Patrick De Clercq

**Affiliations:** ^1^ Department of Crop Protection, Ghent University, Coupure Links 653, B-9000 Ghent, Belgium; ^2^ Current Address: Department of Crop Protection, Vali-e Asr University, P.O.Box 771393641, Rafsanjan, Iran; ^3^ Department of Plant Protection, Islamic Azad University, Takestan Branch P.O. Box: 34819-49479 Takestan-IRAN

**Keywords:** Development, thermal budget, threshold temperature, modelling, biological control, Coccinellidae

## Abstract

The ability of a natural enemy to tolerate a wide temperature range is a critical factor in the evaluation of its suitability as a biological control agent. In the current study, temperature-dependent development of the two-spotted ladybeetle *A. bipunctata* L. (Coleoptera: Coccinellidae) was evaluated on *Myzus persicae* (Sulzer) (Hemiptera: Aphididae) and a factitious food consisting of moist bee pollen and *Ephestia kuehniella* Zeller (Lepidoptera: Pyralidae) eggs under six constant temperatures ranging from 15 to 35° C. On both diets, the developmental rate of *A. bipunctata* showed a positive linear relationship with temperature in the range of 15–30° C, but the ladybird failed to develop to the adult stage at 35° C. Total immature mortality in the temperature range of 15–30° C ranged from 24.30–69.40% and 40.47–76.15% on the aphid prey and factitious food, respectively. One linear and two nonlinear models were fitted to the data. The linear model successfully predicted the lower developmental thresholds and thermal constants of the predator. The non-linear models of Lactin and Brière overestimated the upper developmental thresholds of *A. bipunctata* on both diets. Furthermore, in some cases, there were marked differences among models in estimates of the lower developmental threshold (*t*_min_). Depending on the model, *t*_min_ values for total development ranged from 10.06 to 10.47° C and from 9.39 to 11.31° C *on M. persicae* and factitious food, respectively. Similar thermal constants of 267.9DD (on the aphid diet) and 266.3DD (on the factitious food) were calculated for the total development of *A. bipunctata*, indicating the nutritional value of the factitious food.

## Introduction

In augmentative biological control programmes, detailed information concerning thermal requirements and thresholds is useful for selecting natural enemies that are best adapted to conditions favoring target pests (Jervis and Copland 1996; Obrycki and Kring 1998). During the last decades, numerous linear and non-linear models have been introduced to estimate the thermal thresholds of different species (e.g. Campbell et al. 1974; Stinner et al. 1974; Logan et al. 1976; Sharpe and DeMichele 1977; Lactin et al. 1995; Brière et al. 1999). The linear approximation enables researchers to calculate two constants: the lower developmental threshold (LDT; the temperature below which development is arrested) and the thermal constant or the sum of effective temperatures (SET; the amount of heat needed for completing a developmental stage), within a limited temperature range (usually 15–30° C). However, the non-linear models describe the developmental rate over a wider range of temperatures and provide estimates of maximum and optimum temperatures for development. The weakness of the nonlinear approach is that estimation of SET cannot be achieved and some models do not yield estimates of LDT (Jarošik et al. 2002; Kontodimas et al. 2004).

In order to model insect development as a function of temperature, Logan et al. (1976) developed two empirical models that have been widely used in biological control studies, the so called Logan models 6 and 10 (Roy et al. 2002). Lactin et al. (1995) proposed two modifications of the Logan-6 model, of which the Lactin-2 model allows estimation of the LDT. Brière et al. (1999) proposed two more models with a nonlinear part at low and high temperatures and a linear part at moderate temperatures. In contrast with most of the other models, the Brière-1 and Lactin-2 models enable estimation of all three critical temperatures (minimum, optimum and maximum temperature thresholds). In a number of studies, the Brière-1 and Lactin-2 models proved to be superior at estimating critical temperatures for insects in temperate areas, including coccinellids (Roy et al. 2002; Kontodimas et al. 2004). Further, model application is facilitated when estimation of few fitted coefficients is required. The Brière-1 and Lactin-2 models with three and four coefficients, respectively, as well as the linear model with two parameters are among the models that have the fewest coefficients.

Previous studies have demonstrated the thermal limits of coccinellid predators (e.g. Obrycki and Tauber 1978, 1982; Roy et al. 2002; Mehrnejad and Jalali 2004; Kontodimas et al. 2004). The two spotted ladybird, *Adalia bipunctata* L. (Coleoptera: Coccinellidae) is one of the most common aphid predators occurring in arboreal habitats of Europe, Central Asia and North America (Majerus 1994; Hodek and Honěk 1996). It has potential for the augmentative biological control of aphid pests in Europe (De Clercq et al. 2005). There are a few earlier studies that addressed the thermal requirements of *A. bipunctata* (Obrycki and Tauber 1981; Honěk and Kocourek 1988; Sakuratani et al. 2000). However, the values obtained in all of these studies are only based on the linear model.

The aim of this laboratory study was to estimate thermal requirements and limits for the development of *A. bipunctata* when fed either on a mixture of *Ephestia kuehniella* Zeller (Lepidoptera, Pyralidae) eggs and fresh bee pollen as a factitious food recommended for the rearing of *A. bipunctata* (De Clercq et al. 2005), or on the natural prey *Myzus persicae* (Sulzer) (Hemiptera: Aphididae), using the linear, Brière-1 and Lactin-2 models. The findings of the current study may improve our understanding of the predator's ecology in the field and its phenology in different areas of its distribution. Further, the findings can be indicative for the value of the factitious food for immature development of *A. bipunctata* and as such may contribute to enhancing rearing procedures for this biological control agent.

## Materials and methods

### Predator Culture

Insects were taken from a laboratory colony that was started in August 2002 with eggs purchased from Biobest NV (www.biobest.be); after that, the colony was repeatedly infused with new individuals from the same commercial source. At this commercial facility, the ladybirds had originally been fed with live pea aphids, *A. pisum*, but during this study the stock colony was reared on an ad libitum supply of a 50–50 (w/w) mixture of frozen bee pollen and eggs of *E. kuehniella* (De Clercq et al. 2005). Frozen eggs of *E. kuehniella* and the pollen, consisting of pollen pellets collected by honeybees, used in our study were supplied by Koppert BV (www.koppert.com) and stored for no longer than one month at -18° C. The stock colony of the predator was maintained in a growth chamber at 23 ± 1° C, 65 ± 5% RH and a 16:8 L:D photoperiod.

### Experiments

The study was carried out between September 2004 and March 2005. Experiments were conducted at six constant temperatures (15, 19, 23, 27, 30 and 35 ± 1° C), 65 ± 5% RH and a 16 h photoperiod and using two diets: a mixture of frozen bee pollen and eggs of *E. kuehniella* as a factitious food and live *M. persicae* aphids as a natural food. The aphids were reared on broad bean, *Vicia faba* L. var. *thalia* at 26 ± 2° C, 60 ± 20% RH and a 16:8 L:D photoperiod. For the experiments, a mixture of different nymphal stages of the aphid was provided.

### Development

For the experiment with factitious food, clutches of *A. bipunctata* eggs (<24h old) were collected from the stock colony and distributed among six temperature regimes. At each temperature, at least 50 first instar larvae (<12h old) were isolated in individual 9-cm petri dishes. Each dish contained two plastic cups (3.0 × 0.5cm), one with an ad libitum supply of factitious food and another one with a piece of soaked paper as a water source. Food and water were replenished every one (27, 30 and 35° C) or two days (15, 19, and 23° C).

For the experiments with *M. persicae*, the predator was reared on the aphid prey for one generation at 23° C in order to adapt to the new food source. Eggs from the resulting females were placed in incubators set at 15, 19, 23, 27, 30 or 35° C. At each temperature, at least 50 first instars (<12 h old) were transferred to individual 14-cm petri dishes. Each petri dish was lined with filter paper and contained a 2-leaf seedling of broad bean infested with *M. persicae*. The seedling stalks were inserted in an Eppendorf tube containing water. Aphids were replenished after each daily observation. In each treatment, development and survival of larval and pupal stages were monitored once (at 15, 19 and 23° C) or twice a day (at 27, 30 and 35° C). For calculation purposes, events were assumed to have occurred at the midpoint between two consecutive observations.

### Statistical analysis

Data were checked for normality using the Kolmogorov—Smirnov test (K—S test) and subsequently analysed by Student's t-test. Levene's test was also performed to assess equality of variances. Data were also submitted to two-way ANOVA at α = 0.05 to examine the significance of main effects (food, temperature) and their interaction. Statistical analysis was performed using the SPSS version 15.0 (SPSS 2006) and the JMP version 4.02 (SAS Institute 1989) statistical packages.

### Mathematical Models

Three models were applied to estimate the temperature-dependent development of *A. bipunctata* on *M. persicae* and the factitious food.

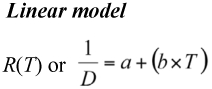

where *R* is the rate of development and *D* is duration of development (days) at temperature *T, a* is the intercept and *b* is the slope of the linear function (e.g. Campbell et al. 1974; Obrycki and Tauber 1982; De Clercq and Degheele 1992; Jarošik et al. 2002; Kontodimas et al. 2004; Mahdian et al. 2008).



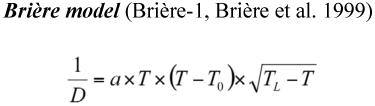

where *T*_0_(*t*_min_) is the lower threshold, *T_L_* (*t*_max_) the lethal temperature (upper threshold) and α is an empirical constant (Brière et al. 1999; Roy et al. 2002; Kontodimas et al. 2004; Arbab et al. 2006, 2008).



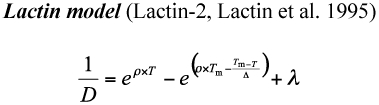

where *ρ*, *T*_m_, *Δ*, and λ are fitted coefficients (Lactin et al. 1995; Lactin and Johnson 1995; Brière and Pracros 1998; Royer et al. 1999; Muniz and Nombela 2001; Tobin et al. 2001; Roy et al. 2002; Kontodimas et al. 2004; Arbab et al. 2006, 2008).

The following indices were calculated, where applicable, for each of the three models:

*The lower developmental threshold* (*t*_min_), defined as the temperature below which there is no measurable development. This value can be estimated by both of the nonlinear models and by the linear model as the intercept value of the temperature axis. The standard error (SE) of *t*_min_, when calculated from the linear model, is:

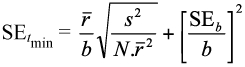

where, *s*^2^ is the residual mean square of *r*, 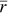 is the sample mean, SE_*b*_, is the standard error of *b*, the slope of linear function, and *N* is the sample size (Campbell et al. 1974; Kontodimas et al. 2004).

*The upper developmental threshold (T_max_)*, defined as the temperature above which the rate of development is zero or life cannot be maintained for any significant period (Kontodimas et al. 2004). This value was estimated only by the nonlinear models, and the SE of *t*_max_ was calculated from the nonlinear regression.

*The optimum temperature for development* (*t*_opt_), defined as the temperature at which the rate of development is maximal. It can be estimated directly from the equations of the nonlinear models and the SE of *t*_opt_ was calculated from the nonlinear regression.

*The thermal constant* (*K*), defined as the amount of thermal energy (degree-days) needed to complete development. The thermal constant *K* can be estimated only by the linear equation:

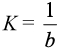

The SE of *K* is (Campbell et al. 1974; Kontodimas et al. 2004):

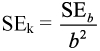



### Fit to data

The following statistical items were used to evaluate the goodness-of-fit: the coefficient of determination (for linear model; *R*^2^) or the coefficient of nonlinear regression (for nonlinear models; *R*^2^) and the residual sum of squares (RSS). Higher values for *R* ^2^ and lower values for RSS reveal better fit.

**Table 1.**
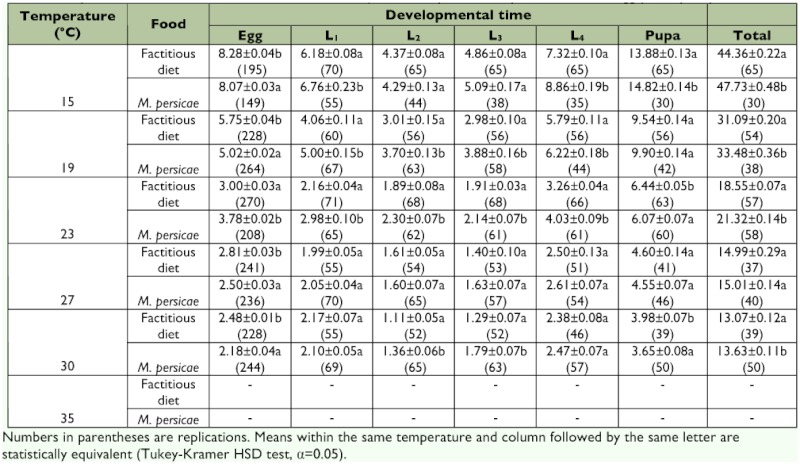
Mean (± SE) developmental time (days) for egg, larval (L_1_ to L_4_), pupal and total immature stages of *Adalia bipunctata* at different temperatures, when fed on either a factitious diet (mixture of pollen and *Ephestia kuehniella* eggs) or *Myzus persicae*.

For each linear regression, the data points at 35° C which deviated from the straight line through the other points were rejected for correct calculation of regression (Campbell et al. 1974; De Clercq and Degheele 1992).

## Results

### Development time

On both diets, the developmental time decreased with increasing temperature from 15 to 30°C (Table 1). The developmental time of the egg stage of the ladybird was more affected by parental food compared to the other developmental stages. A significant difference was observed between the developmental time of the egg stage of *A. bipunctata* on either diet at the range of tested temperatures (t-tests, p<0.001). However, the duration of the larval stage (L1–L4) and egg-adult at 27° C, and of the pupal stage at 19 and 27° C did not differ significantly when the ladybird was reared on either factitious food or on aphid diet (t-tests, p >0.05). A two-way ANOVA for the duration of development of the different immature stages and of total development (egg-adult) with food and temperature as factors revealed a significant interaction of the two factors in all cases (p <0.001).

### Immature survivorship

On both diets, the ladybird successfully developed to the adult stage within the temperature range of 15–30° C. No eggs hatched at 35° C. The highest mortality always occurred in the egg stage and it ranged from 35.9 to 69.6% and from 16.5 to 51.8% on factitious food and aphids, respectively. Overall immature mortality tended to be lowest at low temperatures on the factitious diet, and lowest at intermediate temperatures on the aphid diet (Table 2).

### Thermal constants

Thermal requirements for development of the egg and pupal stages were higher when the predator was fed on factitious food than when fed on aphids but this was compensated by a lower thermal requirement for larval development. Thus, thermal constants for complete development of *A. bipunctata* did not differ between diets (Tables 3 and 4).

### Model evaluation

The results of adjusted *R*^2^ and *RSS* for the different developmental stages of *A.* *bipunctata* and also for total development are presented in Tables 3 and 4. Although the three models yielded a similar *R*^2^, independent of diet, the Lactin model yielded a somewhat lower *RSS* compared to the linear and Brière equations. When *A. bipunctata* was provided with *M. persicae* instead of factitious food, the three models predicted the developmental rate of the predator more accurately, in many cases. For example, for total development time, the Lactin model resulted in a 4% higher *R*^2^ and 80% lower *RSS* value for the aphid diet compared to the factitious food.

**Table 2.**
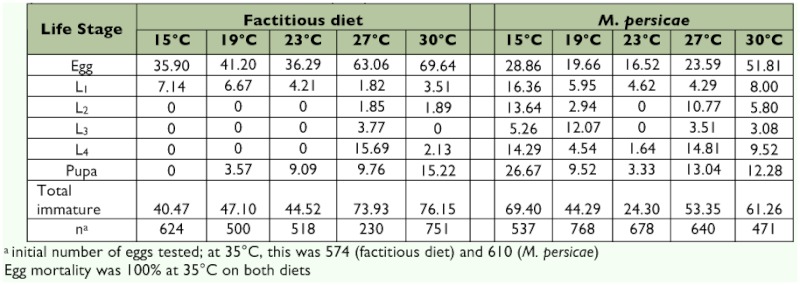
Age-specific mortality (%) of egg, larval (L1–L4), pupal and total immature stages of *Malta bipunctata* at different temperatures, fed on either a factitious diet or *Myzus persicae*.

**Figure 1.**
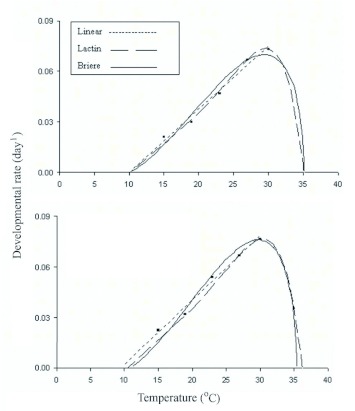
Fitting the linear, Lactin and Brière models to observed values of developmental rates (day^-1^) for egg-adult stage of *Malta bipunctata* as a function of temperature (° C), when the predator was provided with *M. persicae* (above) or factitious food (bottom). High quality figures are available online.

Although the models estimated similar thermal limit values for the total development time of *A. bipunctata* on *M. persicae* (Figure 1), the critical temperatures for pre-imaginal stages of the predator estimated by the Lactin equation were higher than those estimated by the two other models, on both diets (Tables 3 and 4). For instance, the lower threshold for the egg stage on factitious food was estimated to be 8.87 and 9.18° C by the linear and Brière models, respectively. However, the Lactin model predicted it to be 14.79° C. The optimum temperature varied between 29.40 and 33.02° C, depending on developmental stage, diet and the type of non-linear model. Although the experimental data showed that 35° C was lethal for all developmental stages (Table 2), the estimated upper developmental threshold was higher than this value and the employed equations did thus not satisfy the criterion for high model accuracy (Tables 3 and 4).

## Discussion

On both diets, the developmental rate of *A. bipunctata* showed a linear increase with temperature in the range of 15–30°C. Although the average optimum temperature for the different developmental stages was estimated to be in the range of 29.40 to 33.02° C, the mortality data suggested an optimum temperature in the range of 23–27° C. For example, at 30° C the total mortality was 30–45% higher than at 23° C on both diets. A similar result was reported for New York and Ontario populations of *A.* *bipunctata* by Obrycki and Tauber (1981). The latter authors found that the rate of development on *A. pisum* was highest at 29.4° C, but noted that the mean mortality was approximately 25% lower at 26.7° C than at 29.4° C.

All three models fitted the data of the current study well, as indicated by the high *R*^2^ and low *RSS* values, but there were marked differences in the estimated values of *t*_min_ and *t*_max_. A common method for evaluating the accuracy of estimating critical temperatures is based on their comparison with experimental data (Kontodimas et al. 2004). In the current study, the ladybirds successfully developed at the lowest temperature examined (15° C) but failed to develop at 35° C, independent of diet. Therefore, we could not use this criterion but comparison with other studies indicates that this parameter was reliably predicted, especially by the Brière and linear models. In the current study, *t*_min_ values of 10.06 and 9.39° C and thermal constants (*K*) of 267.90 and 266.27 DD were obtained from the linear model for the total development time of *A. bipunctata* on the aphid diet and the factitious food, respectively. The non-linear equations estimated *t*_min_ to be 10.47 and 11.31° C (Brière model) and 8.85 and 7.92° C (Lactin model), on the respective diets. From other published studies, we calculated a *t*_min_ of 8.5° C, and a *K* of 244.8 DD for egg-adult development of a Finnish population of the ladybird fed on *M. persicae* (Ellingsen 1969) and a *t*_min_ of 9.20° C and a *K* of 251.81 DD for a German population fed on *Sitobion avenae* (F.) (Schüder et al. 2004). Also, a similar *t*_min_ of 9.0° C and *K* of 262 DD for the total development of a Nearctic population of *A. bipunctata* on *A. pisum* was reported by Obrycki and Tauber (1981). In central Bohemia (Czech Republic), *t_min_* and *K* values of 10.5 and 8.9° C, and 41.7 and 78.7 DD, respectively, were calculated for development of the egg and pupal stages of the ladybird (Honěk and Kocourek 1988). On the other hand, a lower *t*_min_ of 6.3° C and higher *K* of 322.6 DD for the ladybird was obtained by Sakuratani et al. (2000) in Japan. The latter authors noted that, in this region, *A. bipunctata* is univoltine with adult aestivation and a hibernation diapause from mid June to March.

**Table 3.**
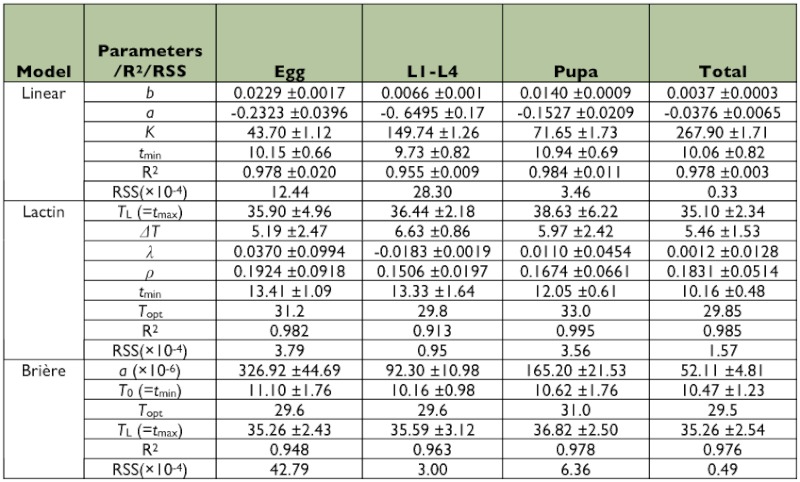
Mean values (± SE) of fitted coefficients and parameters of three developmental rate models describing complete development of *Adelia bipunctata* on *Mysus persicae*.

Although some earlier studies showed that the Lactin and Brière model may be superior for estimating temperature thresholds of ladybird beetles compared to other non-linear models (Roy et al. 2002; Kontodimas et al. 2004), the current study indicates that these models overestimated the upper developmental thresholds of different developmental stages of *A. bipunctata* on the tested diets. In conclusion, the linear model fitted well to the experimental data and should be sufficient for describing temperature dependent development of the coccinellid. The linear model has the advantage of being easy to calculate and is the only model enabling the estimation of the thermal constant (Kontodimas et al. 2004).

To predict the maximum number of generations per year in Western Europe for *A. bipunctata*, we calculated the thermal accumulation for the period of ladybird activity during the year, using the field observations of Hemptinne and Naisse (1987) and the average minimum-maximum temperature data for Brussels from 1971– 2000 (Royal Meteorological Institute of Belgium, www.meteo.be). Based on our estimates of *t*_min_, we predict that *A. bipunctata* starts its spring activity in April when average temperatures are near 10° C and migrates to its hibernation sites in October when average temperatures again approach *t*_min_. Based on thermal requirements estimated in the current study, *A. bipunctata* would produce 3 generations per year. Likewise, Hemptinne and Naisse (1987) stated that in Belgium *A. bipunctata* has 3 to 4 generations through late spring and summer and in autumn adults undergo reproductive diapause until next spring. A similar phenology of *A. bipunctata* was reported by Bazzocchi et al. (2004) in northern Italy.

**Table 4.**
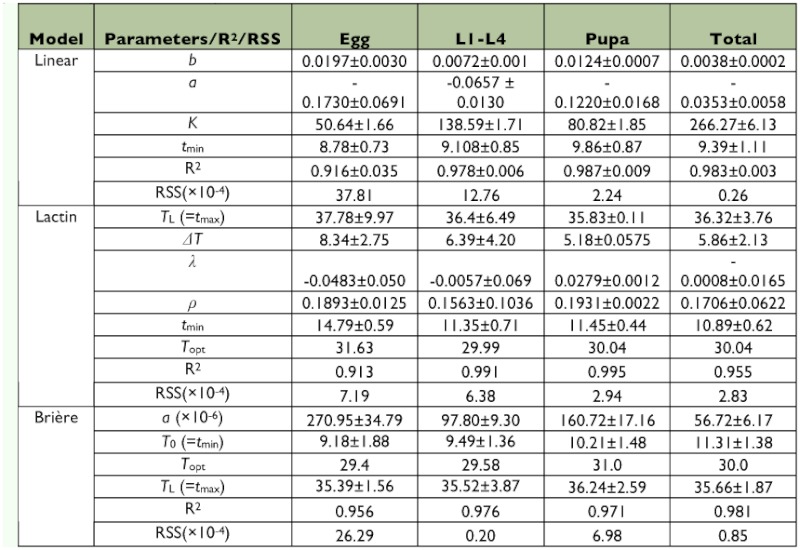
Mean values (± SE) of fitted coefficients and parameters of three developmental rate models describing complete development of *Adalia bipunctata* on the factitious diet.

Certain biotic factors such as food quality and quantity may change the actual number of annual generations of polyphagous predators like *A. bipunctata* in nature. Obrycki and Tauber (1981) proposed that temperature determines the maximum developmental rate of *A. bipunctata* whereas food (availability and type) determines the actual number of generations produced. This is also true for other ladybird species inhabiting temporal regions (Michaud and Qureshi 2006). Food also can directly affect developmental rate (Schüder et al. 2004), length of the preoviposition period and fecundity (El-Hariri 1966), as well as larval development and survival (Blackman 1967) of this ladybird. In our study, food type did not substantially affect thermal requirements of the predator, and larval development was equally successful on the factitious food and the natural prey although in the range of 23–30°C, egg hatch was lower on the factitious food.

Temperature is one of the main ecological factors affecting generation time of a predator and as such determines its ability to track populations of its prey over several generations by its numerical response (Roy et al. 2002). The developmental time of aphidophagous ladybirds like *A. bipunctata* often spans several aphid generations. So the ratio of generation time of these predators to that of their prey is large and a ladybird's rate of increase depends not only on the present state of a patch of prey, but also on the quality of the patch in the future (Kindlmann and Dixon 1999; Dixon 2000; fKindlmann et al. 2007). An important aspect of insect predator-prey dynamics is the difference in the lower temperature thresholds of predator and prey (Dixon 2000). When the predator's lower temperature threshold is substantially higher than the aphid's, the natural enemy is unlikely to have a significant impact on the aphid's abundance, because the predator always arrives too late to prevent aphid population build-up. A review of the literature reveals a much lower *t*_min_ value for *M. persicae* as compared with estimates of *t*_min_ for *A. bipunctata*. Cividanes and Souza (2003) noted that the *t*_min_ and *K* values of *M. persicae* on *Brassica oleracea* L. were 2.2° C and 165.6 DD, respectively., On *Brassica campestris* ssp. *chinensis*, Liu and Meng (1999) calculated *t*_min_ values of 3.9° C and 4.3° C and *K* values of 119.8 DD and 133.0 DD for apterae and alatae forms of *M. persicae*, respectively. Lower developmental thresholds and generation time ratios thus suggest a lack of synchrony between *A. bipunctata* and one of its major prey, *M. persicae*, and would in a classical biological control concept be indicative of a low biocontrol potential of the ladybird. However, pessimistic views on the potential of aphidophagous ladybirds in a classical biological control context may not necessarily be valid for their role in other types of biocontrol (Hodek and Michaud 2008). In augmentative biological control programs using *A. bipunctata*, larval stages (second to third instars) are usually released in relatively high numbers (e.g., Wyss et al. 1999; J. Vermeulen, BioBest NV, personal communication). In an inundative approach usually short term pest suppression by the released larvae and the resulting adults is the objective rather than long term control of the aphid populations. In addition, *A. bipunctata* larvae can be released in aphid hot spots rather than on the entire crop surface.

Our study shows that, irrespective of diet, the developmental rate of *A. bipunctata* linearly increased with temperature from 15 to 30° C, indicating its capacity for predation activity over a wide range of temperatures. Although there is an increase in the use of non-linear models for the prediction of thermal limits of insect natural enemies, our laboratory study shows that a simple linear model may also yield reliable estimates of thermal requirements. Further, similar thermal constants of *A. bipunctata* on the natural prey and a mixture of *E. kuehniella* eggs and pollen corroborate earlier studies indicating the value of latter factitious food for mass production of *A. bipunctata* (De Clercq et al. 2005). Further studies assessing the interaction between climatic factors (e.g. temperature, photoperiod) and food may be helpful in predicting the development of *A. bipunctata* populations in the field and may provide a better understanding of the potential of this predator for biological control of aphid pests.

## Abbreviations

DD: degree day;
*K*: thermal constant or the sum of effective temperatures (SET);
K-S test: Kolmogorov—Smirnov test;
L1 to L4: 1st to 4th instar larvae;
LDT: lower developmental threshold (*t*_min_ or *t*_0_);
RSS: residual sum of squares;
SET: see K;
*t*_0_: see LDT;
*t*min: see LDT;
*T*_L_: upper development threshold;
*t*_max_: see *T_L_*;
*T*_opt_: optimum temperature
